# Using simulation modeling to inform intervention and implementation selection in a rapid stakeholder-engaged hybrid effectiveness-implementation randomized trial

**DOI:** 10.1186/s43058-024-00593-w

**Published:** 2024-06-24

**Authors:** Jessica E. Becker, Fatma M. Shebl, Elena Losina, Anna Wilson, Julie H. Levison, Karen Donelan, Vicki Fung, Hao Trieu, Christopher Panella, Yiqi Qian, Pooyan Kazemian, Bruce Bird, Brian G. Skotko, Stephen Bartels, Kenneth A. Freedberg

**Affiliations:** 1 Department of Child and Adolescent Psychiatry, NYU Grossman School of Medicine, NYU Langone Health, One Park Avenue, Seventh Floor, New York, NY 10016 USA; 2 Medical Practice Evaluation Center, Massachusetts General Hospital, Boston, MA USA; 3 Harvard Medical School, Boston, MA USA; 4 Department of Orthopedic Surgery, Brigham and Women’s Hospital, Boston, MA USA; 5 Mongan Institute, Massachusetts General Hospital, Boston, MA USA; 6 Department of Medicine, Massachusetts General Hospital, Boston, MA USA; 7 Heller School for Social Policy and Management, Brandeis University, Waltham, MA USA; 8 Department of Operations, Weatherhead School of Management, Case Western Reserve University, Cleveland, OH USA; 9 Department of Behavioral Psychology, Kennedy Krieger Institute, Baltimore, MD USA; 10 Down Syndrome Program, Division of Medical Genetics and Metabolism, Department of Pediatrics, Massachusetts General Hospital, Boston, MA USA; 11 Division of Infectious Diseases, Department of Medicine, Massachusetts General Hospital, Boston, MA USA; 12 Division of General Internal Medicine, Department of Medicine, Massachusetts General Hospital, Boston, MA USA

## Abstract

**Background:**

Implementation research generally assumes established evidence-based practices and prior piloting of implementation strategies, which may not be feasible during a public health emergency. We describe the use of a simulation model of the effectiveness of COVID-19 mitigation strategies to inform a stakeholder-engaged process of rapidly designing a tailored intervention and implementation strategy for individuals with serious mental illness (SMI) and intellectual/developmental disabilities (ID/DD) in group homes in a hybrid effectiveness-implementation randomized trial.

**Methods:**

We used a validated dynamic microsimulation model of COVID-19 transmission and disease in late 2020/early 2021 to determine the most effective strategies to mitigate infections among Massachusetts group home staff and residents. Model inputs were informed by data from stakeholders, public records, and published literature. We assessed different prevention strategies, iterated over time with input from multidisciplinary stakeholders and pandemic evolution, including varying symptom screening, testing frequency, isolation, contact-time, use of personal protective equipment, and vaccination. Model outcomes included new infections in group home residents, new infections in group home staff, and resident hospital days. Sensitivity analyses were performed to account for parameter uncertainty. Results of the simulations informed a stakeholder-engaged process to select components of a tailored best practice intervention and implementation strategy.

**Results:**

The largest projected decrease in infections was with initial vaccination, with minimal benefit for additional routine testing. The initial level of actual vaccination in the group homes was estimated to reduce resident infections by 72.4% and staff infections by 55.9% over the 90-day time horizon. Increasing resident and staff vaccination uptake to a target goal of 90% further decreased resident infections by 45.2% and staff infections by 51.3%. Subsequent simulated removal of masking led to a 6.5% increase in infections among residents and 3.2% among staff. The simulation model results were presented to multidisciplinary stakeholders and policymakers to inform the “Tailored Best Practice” package for the hybrid effectiveness-implementation trial.

**Conclusions:**

Vaccination and decreasing vaccine hesitancy among staff were predicted to have the greatest impact in mitigating COVID-19 risk in vulnerable populations of group home residents and staff. Simulation modeling was effective in rapidly informing the selection of the prevention and implementation strategy in a hybrid effectiveness-implementation trial. Future implementation may benefit from this approach when rapid deployment is necessary in the absence of data on tailored interventions.

**Trial registration:**

ClinicalTrials.gov NCT04726371

**Supplementary Information:**

The online version contains supplementary material available at 10.1186/s43058-024-00593-w.

Contributions to the literature

- How can simulation modeling be used to accelerate effectiveness-implementation trials in vulnerable health disparity populations when data on tailored interventions and implementation strategies are limited and the context is urgent and rapidly-evolving?
- A validated microsimulation model of COVID-19 was used to model the potential benefits of mitigation strategies in group homes for individuals with serious mental illness and intellectual/developmental disabilities.
- Model results directly informed a stakeholder-engaged process of selecting key components of a tailored best-practice intervention and implementation strategy in a rapid hybrid effectiveness-implementation trial.
- Simulation modeling was successfully used to inform a rapid effectiveness-implementation trial and has potential to benefit future trial designs.

## Background

A fundamental premise of implementation science is the overarching goal to “bridge the science to service gap” bringing research to clinical action [1–3]. Hybrid effectiveness-implementation research designs may help accelerate the pace of doing so [4, 5], along with stakeholder-engaged tailoring of interventions to improve fit, uptake and scalability [6–9]. Yet, conventional implementation research designs are difficult when the evidence-base is uncertain or rapidly evolving. Additionally, it is challenging to assess the potential merit of a proposed implementation strategy in the context of an urgent public health imperative when time does not allow for pilot studies of feasibility or effectiveness. Methods are needed to select and tailor prevention and implementation strategies. Simulation modeling allows for the ability to inform decisions even when evidence is less robust than would be ideal, by allowing for the examination of assumptions and the impact of uncertainty on the conclusions. The purpose of this report is to describe the use of simulation modeling to rapidly estimate the potential effectiveness of implementing different preventive practices in the context of a public health emergency in a stakeholder-engaged process of tailoring interventions in a randomized effectiveness-implementation trial.

Lacking data on the most effective approaches to preventing COVID-19 in high-risk, vulnerable populations, public health authorities and academic partners were charged with the responsibility to quickly develop and implement preventive practices [10–19]. Residential care, or “group homes,” for adults with serious mental illness (SMI) and intellectual and developmental disabilities (ID/DD) represent particularly high risk settings and populations for high rates of adverse outcomes associated with COVID-19 [19–22]. Individuals with SMI and with ID/DD in group homes (GHs) are at especially high risk due to close proximity; increased rates of risk factors such as obesity, smoking, metabolic disease, and cardiovascular disease; and challenges in adopting safe health practices such as mask use, social distancing, and vaccination [23–38]. Finally, adults with disabilities, including SMI and ID/DD, represent a vulnerable health disparity population prior to the pandemic given their reduced life expectancy.

Responding to the need to simultaneously implement and study the effectiveness of different COVID-19 mitigation strategies, we deployed a validated microsimulation model of COVID-19 transmission to identify “tailored best practices” for the population of residents and staff in group homes for adults with SMI and ID/DD. This microsimulation model was used to produce specific estimates of the impact of different approaches to COVID-19 risk mitigation to inform a stakeholder-engaged process of selecting and tailoring the final intervention and implementation process for a hybrid effectiveness-implementation trial. A comprehensive description of the hybrid effectiveness implementation study protocol is reported elsewhere [39].

## Methods

### Overview

The primary aim of the parent randomized trial is to evaluate the effectiveness and implementation of General Best Practices (GBP) compared to Tailored Best Practices (TBP) in preventing COVID-19 in the staff and residents of group homes for adults with serious mental illness (SMI) and intellectual and developmental disabilities (ID/DD) [39]. General Best Practices (GBP) reflected general guidelines for COVID-19 prevention provided by the Massachusetts Department of Health and Human Services and the Center for Disease Control. Tailored Best Practices (TBP) consisted of COVID-19 prevention practices specifically selected to address the risk factors, group home settings, and populations of residents with SMI and ID/DD. The process of selecting the core components of TBP was a collaborative co-design process with stakeholders including SMI and ID/DD residents, group home providers, community service organization representatives and leaders, and advocacy groups. The multidisciplinary, stakeholder-driven process was informed by a validated mathematical simulation model of COVID-19 disease, the Clinical and Economic Analysis of COVID-19 interventions (CEACOV) model, to project the clinical impact of a variety of different potential COVID-19 mitigation strategies in the staff and residents with SMI and ID/DD in group homes.

The CEACOV model is a dynamic microsimulation model of the natural history of COVID-19 disease and utilizes a susceptible, exposed, infectious, recovered (SEIR) framework [40, 41]. The model is publicly available, as detailed in the Supplement. In this application of the model, three defined population groups were assessed including residents of GHs, staff of GHs, and the surrounding community mixed in a heterogeneous manner. New cases were transmitted at a probability resulting from a combination of the virus infectivity rate, likelihood of contacting others both within and outside the population group, number of other individuals contacted, and length of contact time. Additional details of the model design and structure are published elsewhere and included in the Supplement [40, 41].

The model inputs for this analysis were derived to mirror the population of interest for the anticipated hybrid effectiveness-implementation trial consisting of the residents and staff of 415 GHs for individuals with SMI and ID/DD in Massachusetts. The model inputs reflect the population in late February-early March 2021 during trial planning; given the rapidly-evolving nature of the COVID-19 pandemic at the time, the time horizon for model analysis was 90 days. Outcomes included number of new COVID-19 infections in GH residents, number of new COVID-19 infections in GH staff, and total number of hospital-days among GH residents. The model has been validated against Massachusetts data and in similar congregate living settings [40, 42]. To do so, the model was calibrated to transmission data spanning various 30-day intervals and used to predict the outcomes over the following 15 days. We used the reported number of COVID-19-related deaths as the primary calibration target. To assess model fit, we used the mean absolute percentage error (MAPE) and the median absolute percentage error (MEDAPE) for modeled and observed number of deaths over the validation horizon [42].

The study was approved by the Institutional Review Boards of MassGeneral Brigham, the Massachusetts Department of Developmental Services, and the Massachusetts Department of Mental Health. A review of our stakeholder groups, data source types, and model input derivations follow; additional details of each can be found in the Supplement.

### Stakeholder groups

**Fig. 1:**
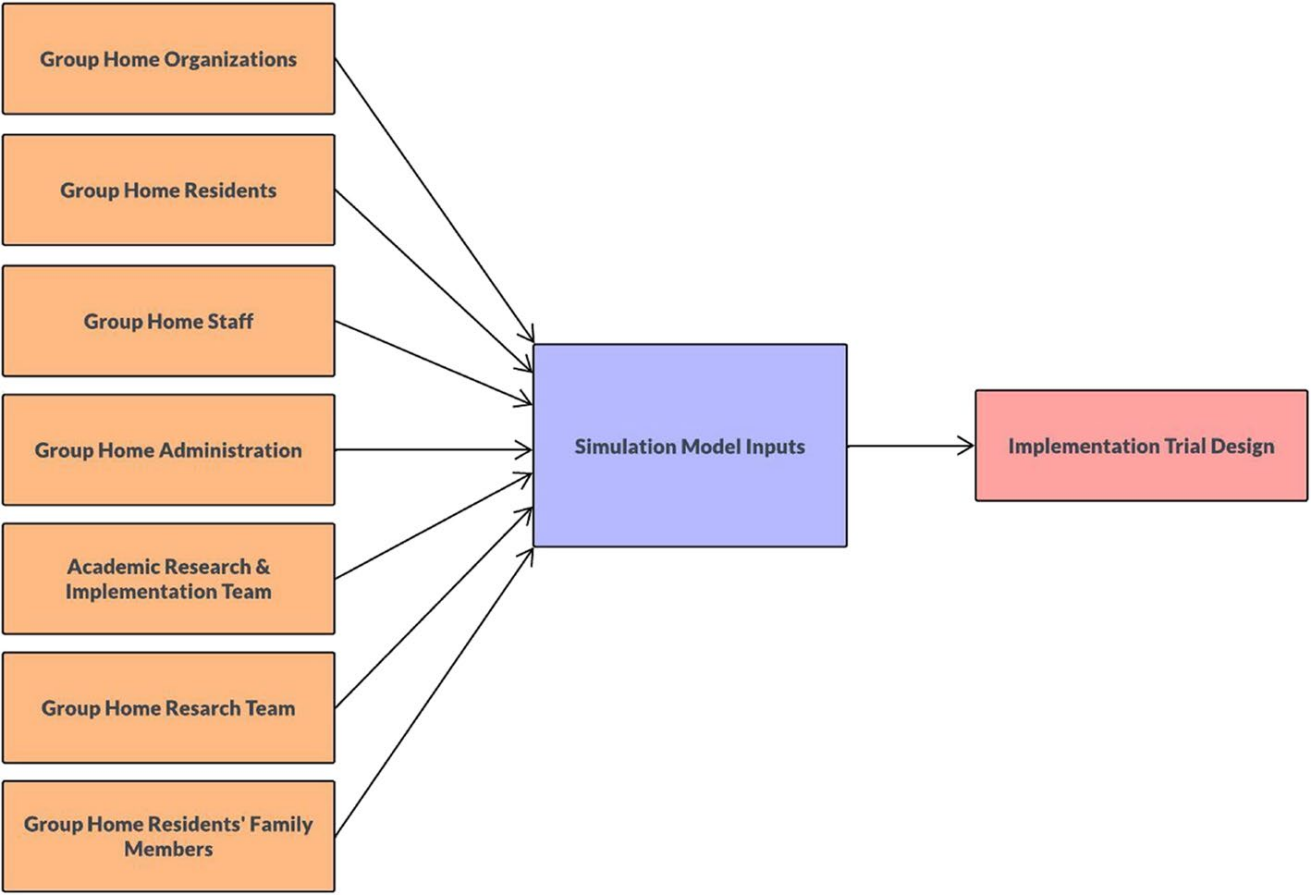
Stakeholders for simulation modeling to inform an implementation trial of best practices to mitigate COVID-19 disease in group homes for individuals with serious mental illness and intellectual disability/developmental disability in Massachusetts. This figure depicts the multiple stakeholder groups with whom the simulation modeling team collaborated in order to derive model inputs reflective of the population of interest, namely the residents and staff of a sample of 415 group homes in Massachusetts

As described in the detailed study protocol [39], project-based partnerships with stakeholders were instrumental in determining and obtaining model inputs and designing the strategies examined in the model analyses. Figure 1 highlights the various stakeholders that were included in designing and carrying out the model analyses. Additional details of the role of each stakeholder group to the modeling analysis can be found in the Supplement (eTable 1).

### Data sources

Data to inform model inputs were derived from four source types: qualitative data, including the design and use of a survey and structured qualitative interviews, obtained from GH residents and staff in partnership with stakeholders; quantitative data collected in collaboration with the GH research team and administration; publicly available data from the Massachusetts Department of Public Health; and published literature. Additional details about how these data sources relate to input parameters can be found in the Data Sources section of the Supplement (Supplement page 2). The entire structure of the model, for further clarification along with the data sources above, is available online at https://github.com/MGH-MPEC/CEACOV.

### Input parameters

Model input parameters in the simulation reflected the unique trial GH population, including residents and staff (Table 1).

**Table 1:** Input parameters for an analysis of COVID-19 mitigation strategies in congregate living facilities for individuals with serious mental illness and intellectual/developmental disabilities in Massachusetts

**Parameter**	**Value**			**Source Category**	**Sources**
**Cohort characteristics**					
	Residents	Staff	Community		
Baseline cohort distribution across transmission groups	0.032	0.044	0.924	Group Homes	Provider Organizations
Baseline age distribution, %				Group Homes	
< 20	0	0	0.2		Provider Organizations, Assumption (community)
20-59y	0.718	0.9	0.6		
≥ 60y	0.282	0.1	0.2		
Initial proportion susceptible, pre-vaccination	0.91998	0.91998	0.91998	State	Derived from Massachusetts Department of Public Health [43–45]
**Infectivity & Contacts**					
Infectivity/contact/hour	0.002			Literature	Losina et al.,* CID 2020;* derived from Li et al., *CID* 2020 [41, 46]
**Adherence to Non-pharmacologic Interventions**					
*Base case*					
	Residents	Staff	Community		
Mask adherence in group home	0.244	0.9	–	Group Homes	Provider Organizations
Mask adherence in personal home	–	0	0		Assumption
Mask adherence in community	0.764	0.764	0.764	Literature	Fisher et al., *MMWR*, 2020 [47]
Adherence to Residence Isolation	0.5	0.5	0.5		Assumption
**Vaccine Characteristics**					
Efficacy in preventing symptomatic illness		0.95		Literature	Polack et al., *NEJM* 2020 [48]
Efficacy in preventing any infection		0.8			Assumption
Efficacy in preventing symptoms if infected after vaccination		0.75			Assumption
	Residents	Staff	Community		
Vaccination Uptake	0.72	0.58	0.05	Group Homes	Provider Organizations, Assumption
**Test characteristics**					
Sensitivity (day of infection)					
1–4	0.165			Literature	Losina et al. 2020 [41]; Kucirka et al. 2020 [49]
5–9	0.71				
10–21	0.435				
> 22	0				
Specificity, %	100				

#### Cohort

Using baseline data collected from the GHs, including the total populations of 3,469 residents and 4,797 staff across all GHs, an average GH size of 7 residents, 3 staff shifts per day, and average staff:resident ratios, we modeled the cohort of residents and staff at all 415 participating GH sites, along with an estimated 100,000 individuals in the surrounding community. Given that staff sometimes worked at more than one GH, and that residents often shared day programming with residents of other GHs, we modeled all participants together. To account for potential stochastic variation in a relatively small model simulation cohort size, we used model runs with 10 times the number of total individuals across the GHs and scaled results down by a factor of 10 to adjust for the study cohort size. Cohort characteristics, such as gender and age, reflected data gathered from the GH organizations on GH residents and staff; community demographic data were based on publicly-available data. The proportion susceptible to COVID-19 infection was calculated using publicly-available data from Massachusetts in late February-early March 2021, including the total number of active cases, total cases, total deaths, and total population across the Commonwealth. In an effort to generate conservative estimates prior to understanding the full extent of the pandemic on specific population subgroups, the initial proportion susceptible was assumed to be the same across the three population groups.

#### Contact-hours and infectivity

To understand the dynamics and interactions of residents and staff within and outside the GHs, we used data collected by survey and stakeholder interview to estimate the number of contact-hours for each transmission group in the first year of the pandemic. The force of infection from each transmission group was derived utilizing an overall infectivity per contact-hour weighted by contact-hours spent with each other transmission group, capturing the heterogeneity of viral transmission across the population [41, 46, 50]. Since the horizon of the analysis was 90 days, we did not model waning immunity or reinfections.

#### Mitigation interventions

Mitigation interventions, including COVID testing frequency, isolation, and resident mask adherence, were modeled to reflect actual practice in the GH organizations in the planned trial as determined through survey data. Modeling of mask efficacy, staff and community mask adherence, and staff and community isolation were informed by prior studies [41, 47, 51]. The impact of these interventions on force of infection in the model is described further in the Input Derivation section of the Supplement.

### Strategies

An ongoing challenge for the intervention and implementation process to prevent COVID-19 in the group homes was an evolving population incidence rate of COVID-19 and changing evidence base and recommendations for effective preventive practices. Evolving changes in this outer context required ongoing dynamic adaptations and revisions in the components of the intervention and implementation strategies consistent with agile implementation methods and dynamic sustainability. We revised our examined prevention strategies over time to reflect the rapid progression of the COVID-19 pandemic, new knowledge on prevention, and real-time input from stakeholders around shifting abilities of the GHs to support mitigation measures. In the initial early planning stages, preventive interventions included different frequencies of routine testing, off-site isolation of quarantining residents, and staffing changes. However, in discussions with stakeholders, it was determined that these administrative changes were not feasible to be evaluated in a randomized trial. As such, they were not included in the final examined prevention strategies. At the same time, the development and approval of vaccinations in the United States during the planning stages of the implementation trial required a course shift and rapid incorporation of vaccination into the model that was not present as an option in the early planning stages of the study [48].

The final, base case model prevention strategies in the simulation, which were used to help guide decision-making of stakeholders in selecting tailored best practice interventions for the trial, consisted of the following:

1. *Early Pandemic:* This early prevention strategy reflected mitigation measures used in the earliest days of the COVID-19 pandemic, with reduced mobility as the sole mitigating measure and hospitalization available for severe COVID-19 disease.
2. *Early Pandemic with Masks:* This early prevention strategy reflected the dynamics of incorporating mask use among residents, staff, and community members; reduced mobility; and hospitalization for severe COVID-19 disease during the early stages of the COVID-19 pandemic (March-December 2020).
3. *Pre-vaccination Status Quo:* In addition to the measures included in the Early Pandemic with Masks prevention strategy, this prevention strategy also included the GH staff and resident symptom screening, asymptomatic testing among staff in GHs, isolation, and contact-time reflecting the standard practices in our GH population just prior to the introduction of vaccines in early 2021.
4. *Vaccination Current Levels:* In addition to all measures included in the Pre-vaccination Status Quo strategy, this prevention strategy reflected the addition of vaccination uptake in 72% of residents and 58% of staff, reflecting population vaccination levels current during the analysis in early March 2021 after the initial wave of vaccinations in the GH study group.
5. *Vaccination Target Uptake:* In addition to all measures included in the Vaccination Current Levels strategy, this prevention strategy reflected the target vaccination uptake of 90% both in the population of GH residents and staff in March 2021.

This combination of strategies allowed for assessment of the projected impact of individual prevention measures, layering from one strategy to the next and including those already put in place, to help stakeholders decide which particular prevention measures would be most feasible and impactful to include in the TBP package for the hybrid effectiveness-implementation trial. Quantifying the estimated benefit of specific measures already in place provided stakeholders with additional insight into whether to emphasize such strategies moving forward as part of the TBP package.

Given the large impact of vaccination on reducing infection rates, vaccination uptake was subsequently varied incrementally among residents and staff, both separately and together. Vaccination was also examined with regard to the hiring of new, unvaccinated staff (assumed 25% staff turnover per year in discussion with stakeholders); scale-up from biweekly to weekly asymptomatic testing in GH staff and from symptomatic-only to weekly asymptomatic testing in GH residents; and mask removal.

### Outcomes

The primary outcomes of the modeling analysis were new infections in GH residents and new infections in GH staff. The secondary outcome was resident hospital days. Multiple sensitivity analyses were performed, including varying vaccine efficacy in preventing any infection and vaccine efficacy in preventing symptomatic infection.

### Planned translation of results to the trial

At the project outset, the process for identifying the intervention components of “Tailored Best Practices” was to use a stakeholder-engaged approach with the plan to transmit the plans for the simulation to stakeholders as part of a stakeholder summit for the trial, the COVID Quality Improvement Collaborative (CQIC) Summit [39]. Results were subsequently presented to stakeholders, including the project’s research team, data analyst, and GH provider organization leadership, on both a scheduled and ad hoc basis.

## Results

### Modeling results

#### Base case prevention strategies prior to vaccine availability

The base case *Early Pandemic* prevention strategy resulted in 983 new infections in residents and 2,588 new infections in staff over a 3-month period, along with a projected 72 resident hospital-days (Table 2). The *Early Pandemic with Masks* prevention strategy incrementally reduced resident infections by 52.1% to 472, staff infections by 44.7% to 1,430, and resident hospital-days by 40.3% to 43 over the 3-month period. The *Pre-vaccination Status Quo* prevention strategy led to a further 35.5% reduction in resident infections to 304, a reduction in staff infections by 17.6% to 1,178, and a reduction in resident hospital-days by 37.2% to 27.

**Table 2:** Results of an analysis of initial management strategies over 90 days for COVID-19 prevention in group homes for individuals with serious mental illness and intellectual/developmental disabilities in Massachusetts

**Strategy**	**New Infections (n, %)**		**Resident Hospital Days**
	**Residents** ***N* = 3,469**	**Staff** ***N* = 4,797**	
*Early Pandemic*	983 (28%)	2588 (54%)	72
*Early Pandemic with Masks*	472 (14%)	1430 (30%)	43
*Pre-vaccination Status Quo*	304 (9%)	1178 (25%)	27
*Vaccination Current Levels*	84 (2%)	519 (11%)	18
*Vaccination Target Uptake*	46 (1%)	253 (5%)	6

#### Base case prevention strategies with vaccination

The greatest incremental decrease in infections in the model was with the *Vaccination Current Levels* strategy; resident infections dropped by an additional 72.4% to 84 over the 3-month period, while staff infections dropped another 55.9% to 519; resident hospital-days dropped by one-third to 18. Under the *Vaccination Target Uptake* strategy, resident infections dropped by another 45.2% (84 to 46), staff infections dropped by another 51.3% (519 to 253), and resident hospital-days dropped by another 66.7% (18 to 6) compared to the *Vaccination Current Levels* strategy.

#### Additional testing and vaccination strategies

We then examined prevention strategies with increased testing frequency, staff turnover, and vaccination uptake (Table 3). Adding weekly staff and resident COVID-19 testing to the *Vaccination Current Levels* prevention strategy was projected to have minimal impact on resident and staff infections, with only a 1.2% drop in each; this strategy was associated with a 27.8% drop in resident hospital-days, likely attributable to the impact of identification and subsequent isolation linked to an asymptomatic positive test result among residents. At the *Vaccination Current Levels*, adding in staff turnover resulted in a 4.8% increase in resident infections, a 2.3% increase in staff infections, and a 16.7% increase in resident hospital-days.

**Table 3:** Results of a modeling analysis of vaccination strategies for COVID-19 prevention in group homes for individuals with serious mental illness and intellectual/developmental disabilities in Massachusetts

**Strategy**	**New Infections**		**Resident Hospital Days**
	**Residents (*N* = 3,469)**	**Staff (*N* = 4,797)**	
*Pre-vaccination Status Quo*	304	1178	27
*Vaccination Current Levels*	84	519	18
*Vaccination Current Levels with weekly staff and resident tests*	83	513	13
*Staff Turnover*	88	531	15
*Resident Vaccination Uptake*			
80%	70	518	4
90%	51	515	12
*Staff Vaccination Uptake*			
70%	76	419	13
80%	76	325	10
90%	67	249	10
*Resident and Staff Vaccination Uptake:*			
80%	64	327	3
90%	46	253	6
90% with no masks	49	261	7

**Fig. 2:**
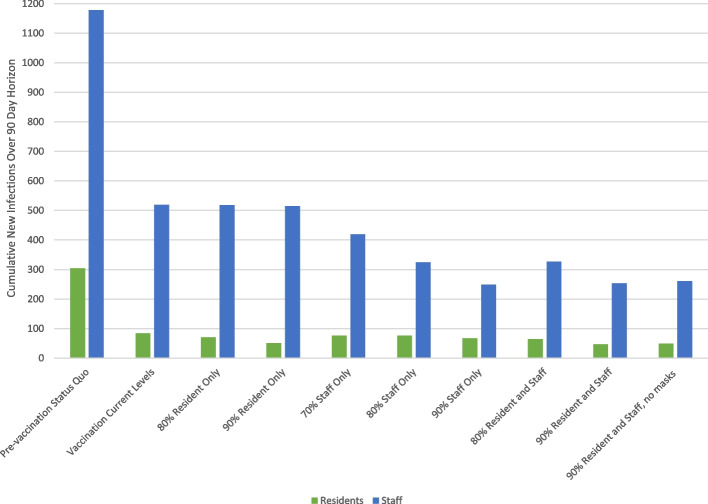
Total new infections over 3-month projection, grouped by vaccination group. This figure displays the projected cumulative number of new COVID-19 infections among residents and staff of 415 group homes for individuals with serious mental illness and intellectual disability/developmental disability in Massachusetts under a variety of modeled vaccination strategies, over the 90-day modeled time horizon. Model input parameters are informed by data from the group homes, as well as Massachusetts publicly-available public health data and the published literature. Projected cumulative infections in the setting of vaccination levels current at the time of the model analysis are displayed, as well as strategies with staff turnover and increased resident and staff vaccination uptake. Given the lower baseline vaccine uptake among staff, increasing staff uptake makes the largest incremental difference in preventing both resident and staff infections

**Fig. 3:**
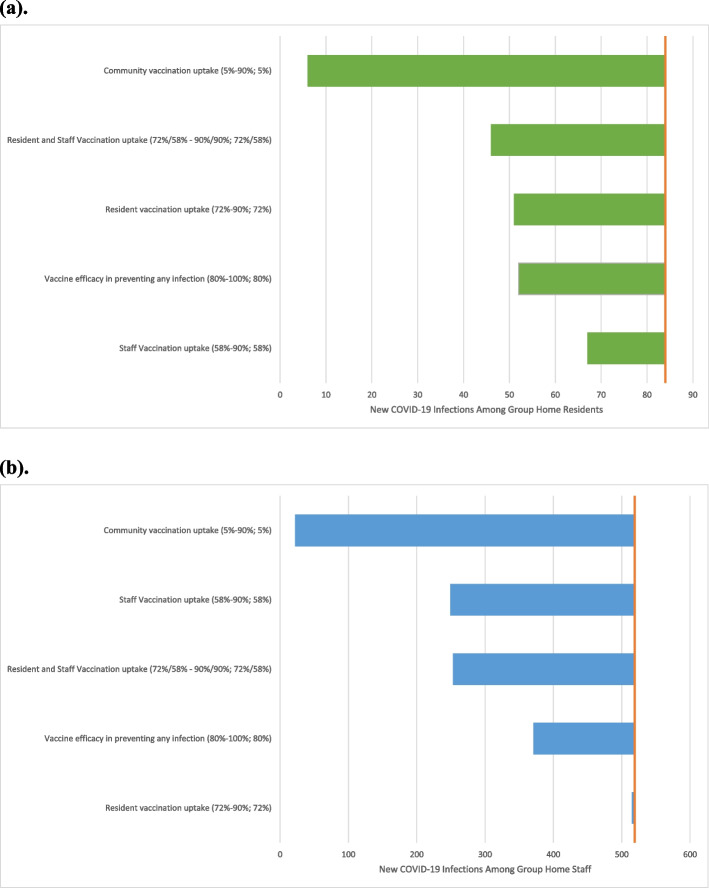
Factors affecting development of new infections among residents and staff of group homes for individuals with serious mental illness and intellectual disability/developmental disability in Massachusetts. This figure displays the projected impact on the of varying uncertain input parameters, including vaccine efficacy and vaccination uptake among residents, staff, and community members, on the development of new COVID-19 infections among **a** residents and **b** staff of group homes for individuals with serious mental illness and intellectual/developmental disability in Massachusetts included in a hybrid implementation-effectiveness trial. The values of each parameter are indicated in parentheses in the following format: (smallest value examined - largest value examined; value in the base case *Vaccination Current Levels* strategy). The orange line in (**a**) indicates the number of projected resident infections (84) for the base case *Vaccination Current Levels* strategy. The orange line in (**b**) indicates the number of projected staff infections (519) for the base case *Vaccination Current Levels* strategy. The results summarized in the figure helped to inform targets of the trial’s tailored best practice intervention package for COVID-19 risk mitigation

We then examined the impact of increasing vaccination uptake among residents alone, staff alone, or residents and staff together (Table 3, Fig. 2). Increasing staff vaccination uptake had the largest impact on decreasing both resident and staff infections over the 3-month time horizon, while increasing resident vaccination uptake alone had less impact. When incrementally removing mask use among the population of residents and staff with 90% vaccination uptake, resident and staff infections increased slightly. Results were robust to sensitivity analyses examining vaccine efficacy in preventing any infection and vaccination uptake in residents, staff, and community members (Fig. 3).

### Translation of results

The modeling team presented simulation plans to GH residents, staff, family members, and administrative leadership along with the full research and implementation trial teams at the CQIC Summit in February 2021. At this Summit, the broader study team presented to all stakeholders both qualitative and quantitative data derived from the GHs in the study sample, which ultimately informed the modeling inputs, including demographics, early infection data, precaution measures taken, and perceived impact of the pandemic on daily function. Over the subsequent months in scheduled and ad hoc meetings, stakeholders were provided an overview of the simulated impact and estimated additional benefits of the different prevention measures and weighed the comparative benefits of different measures relative to the practical feasibility of implementing the intervention components into the trial. The modeling results thus directly informed the selection of the key components of the TBP intervention incorporated into the implementation trial. For example, the relatively high resource need associated with routine testing was considered to be outweighed by the benefits of mask use and vaccination. Similarly, despite the improved outcomes predicted with use of masks, it was recognized that this was highly feasible and enforceable for staff, but challenging for individuals with SMI and ID/DD as a mandate in their living environment. Simulation modeling demonstrating a small incremental benefit of pursuing adherence to wearing masks for residents with SMI and ID/DD beyond vaccination confirmed for the stakeholders that the substantial effort required to reinforce this behavior for residents would be better dedicated to behavioral change efforts promoting vaccination. Implementation strategies then prioritized immunization for residents and staff, while at the same time promoting vaccinations and strict adherence to mask use by staff. Model results highlighting the expected impact of vaccine uptake on infections in the GHs were also used in the educational materials used with GH residents and staff as a component of the TBP intervention (Supplement eFigure 1).

The simulation modeling results were also presented directly to governmental policymakers, including stakeholders from the Massachusetts Department of Mental Health and Massachusetts Department of Developmental Services. The results of the simulation modeling, particularly around the impact of vaccination for the GH population, were used to advocate for prioritizing GH residents and staff in the statewide vaccine distribution at the time of limited and restricted initial vaccine availability.

## Discussion

This study demonstrated the feasibility and potential benefits of applying methods of simulation modeling coupled with a stakeholder-engaged process to rapidly select multi-component, tailored, targeted, intervention and implementation strategies in a hybrid effectiveness-implementation research trial. This approach was developed and applied in the context of a public health emergency in which the incidence of infection and the evidence base for effective preventive practices was evolving, and the target population consisted of a health disparity population at high-risk for adverse outcomes with unique vulnerabilities and capacities.

In the specific context of developing and evaluating best practices to prevent COVID-19 infection in the staff and residents of group homes, our modeling results demonstrated the positive impact of mitigation measures already in place in the GHs and showed that increasing vaccine uptake would be the most efficacious next target for the trial. We found the incremental benefit of mask use in GHs was substantially diminished in the context of effective vaccination, which was of particular significance in the population of adults with SMI and ID/DD in GHs, as efforts to achieve and sustain full compliance with the use of face masks and social distancing was challenging, and in many instances, not possible. In contrast, focusing efforts on implementation strategies aimed at improving resident and, particularly, staff vaccination uptake was shown in simulation to have the maximum potential benefit for both residents and staff. Identifying this focus as an implementation target was underscored by the lower proportion of staff, compared to residents, who were vaccinated at baseline. While there is little, if any, literature on successful COVID-19 mitigation in GH settings for vulnerable adults with SMI and ID/DD, this finding is similar to that found in a modeling analysis examining mitigation practices in nursing homes, a setting with residential and staff-support functions similar to those in GHs [52].

Conventional implementation research usually relies on the existence of a robust evidence base with the primary goal of identifying an optimal strategy for implementing well-documented, effective practices. In contrast, hybrid effectiveness-implementation trials simultaneously seek to identify the most effective interventions and optimal implementation strategies. In this instance investigators develop hypotheses by the potential components and benefits of a given practice based on preliminary pilot data (if these exist) or extrapolating and adapting related data. In the COVID-19 pandemic, public health officials and researchers were challenged to quickly develop and implement recommendations for special high-risk populations in the absence of precedent. Our investigation demonstrates the value of using microsimulation modeling to inform the prioritization and selection of interventions and implementation strategies in settings serving vulnerable populations for rapid deployment in a public health emergency. Moreover, simulation modeling offers a way to estimate the effectiveness of interventions under novel conditions. As such, we propose that modeling may be useful to inform implementation and hybrid effectiveness-implementation trials even beyond the scope of an emergency and when a greater evidence base is available, to help stakeholders better estimate anticipated costs and benefits and thus prioritize among potential interventions for a trial in a more structured, reliable way.

A limited existing literature supports the use of mathematical simulation modeling to inform the design of research trials, albeit primarily clinical trials [53–57]. While recent literature has highlighted the potential value of using simulation modeling in the design of implementation trials [58–60], there is a lack of data demonstrating the feasibility and actual use of simulation modeling in the design of implementation research trials, especially for the design of hybrid effectiveness-implementation trials. Sheldrick et al. recently suggested guidelines to inform the use of simulation modeling in implementation – a process they named rapid-cycle systems modeling (RCSM) [60]. While our team began this project prior to the publication of the RCSM framework, our goals and process corresponded to that recommended in RCSM, including determining stakeholders’ needs, refining a simulation model, and utilizing stakeholder input to iterate modeling [60]. Huang and colleagues also recently describe the use of agent-based and microsimulation models to inform implementation trials [59]. Our work builds on that analysis by adding networking and incorporating a disease transmission probability, allowing us to examine the spread of an infectious disease in the population. Moreover, Huang et al. detail the process of developing a new model, which can take substantial additional time and resources when designing an implementation trial [59]. In contrast, our study benefited from expansion of an existing, validated simulation model, which allowed both for accuracy and rapidity of iterations in the context of the fast-moving pandemic [40–42, 61–63].

Taken together, our analysis and these studies support the feasibility and potential major benefits to utilizing simulation modeling in implementation trial design. We suggest several practices for such future use. First, simulation modeling partnering with stakeholders allows for the use of stakeholder-specific data to project anticipated results for the population and setting of interest. Given that simulation models often rely primarily on the published literature for data, model results may be limited in their generalizability. Alternatively, by partnering with stakeholders directly and collecting data from the source that an implementation study is targeting, modelers can devise and obtain the input parameters needed and address the stakeholders’ questions of interest for that specific population. Second, our study demonstrates the use of simulation modeling coupled with stakeholder-engaged co-design to inform the identification of the most promising and feasible prevention strategies for implementation. Utilizing this process in future implementation studies may allow for more efficient resource use in implementation research trials, by better narrowing in on the key components of interventions expected to have effective results prior to launching large-scale trials. Third, the results of stakeholder-informed simulation modeling can be used to communicate outcome data in real-time to stakeholders. In this project, modeling results were used in educational materials as part of the tailored best practice intervention; GH staff, residents, and administration were able to learn insights directly from their own data, which helped with buy-in and subsequent tailoring of interventions. Fourth, the process of revising and refining the selected prevention strategy in collaboration with stakeholders to make real-time changes to simulation model strategies and data inputs allowed for refined recommendations for our population of interest. Future implementation studies can also benefit from rapidly-iterated, data-informed simulation model outcomes, particularly in rapidly-changing contexts, exemplified in this study in the rapidly-evolving COVID-19 pandemic. Finally, given that the Food and Drug Administration and other similar agencies look for evidence on plausible mechanisms of action in reviewing applications, simulation modeling of implementation science trials could be used as a part of that process [64].

This study has several limitations. First, given the goal of informing an implementation trial in Massachusetts, the modeling results may not have been generalizable to other settings. However, we believe that the GH-specific data, which we obtained by partnering with stakeholders, may be informative to similar facilities for individuals with SMI and ID/DD across the country, given the paucity of data for this population in the published literature. Second, we scaled up the number of participants and scaled down our results accordingly in order to account for potential statistical noise in the model results with a small sample size; however, in doing so, some transmission dynamics could have been changed and the structural details of particular GHs may not have been fully accounted for. However, the participation of the research and GH administrative staff allowed for direct communication with the implementation teams about the modeling methodology and interpretation of the results.

## Conclusion

We used a mathematical simulation model of COVID-19 disease to rapidly estimate the potential effectiveness of implementing different preventive practices in GHs, in response to a public health emergency and to inform a randomized effectiveness-implementation trial. The results of the simulation modeling confirmed the primary importance of prioritizing vaccination combined with masking for staff in reducing COVID-19 infections in GH settings. In addition, these simulations provided critical data to guide the tailoring of the final intervention and implementation process to the unique context of group homes and the health disparity population of residents with SMI and ID/DD. We also established the successful use of a modeling-implementation partnership to optimize the implementation trial design by using simulation modeling to inform the stakeholder-engaged process of selecting the key components of the intervention and implementation strategies. This paradigm of pilot modeling can accelerate the process and improve the efficiency and quality of future implementation research trials, including in a future public health emergency.

### Supplementary Information

Supplementary Material 1.

## Data Availability

The simulation model developed by our research team and used in this analysis, the Clinical and Economic Analysis of COVID-19 interventions (CEACOV) model, is publicly-available at the following website: https://github.com/MGH-MPEC/CEACOV. The data generated/analyzed during this study are available from the corresponding author on reasonable request.

## References

[1] Chambers DA (2018). Commentary: increasing the connectivity between implementation science and public health: advancing methodology, evidence integration, and sustainability. Annu Rev Public Health.

[2] Westerlund A, Sundberg L, Nilsen P (2019). Implementation of implementation science knowledge: the research-practice gap paradox. Worldviews Evid Based Nurs.

[3] Bauer MS, Kirchner J (2020). Implementation science: what is it and why should I care?. Psychiatry Res.

[4] Curran GM, Bauer M, Mittman B, Pyne JM, Stetler C (2012). Effectiveness-implementation hybrid designs: combining elements of clinical effectiveness and implementation research to enhance public health impact. Med Care.

[5] Curran GM, Landes SJ, McBain SA (2022). Reflections on 10 years of effectiveness-implementation hybrid studies. Front Health Serv.

[6] Goodman MS, Sanders Thompson VL (2017). The science of stakeholder engagement in research: classification, implementation, and evaluation. Transl Behav Med.

[7] Concannon TW, Meissner P, Grunbaum JA (2012). A new taxonomy for stakeholder engagement in patient-centered outcomes research. J Gen Intern Med.

[8] Aarons GA, Hurlburt M, Horwitz SM (2011). Advancing a conceptual model of evidence-based practice implementation in public service sectors. Adm Policy Ment Health Ment Health Serv Res.

[9] Concannon TW, Fuster M, Saunders T (2014). A systematic review of stakeholder engagement in comparative effectiveness and patient-centered outcomes research. J Gen Intern Med.

[10] Hamada K, Fan X (2020). The impact of COVID-19 on individuals living with serious mental illness. Schizophr Res.

[11] Chen S, Jones PB, Underwood BR (2020). The early impact of COVID-19 on mental health and community physical health services and their patients’ mortality in Cambridgeshire and Peterborough. UK J Psychiatr Res.

[12] Fond G, Pauly V, Leone M (2021). Disparities in intensive care unit admission and mortality among patients with schizophrenia and COVID-19: a national cohort study. Schizophr Bull.

[13] Fond G, Pauly V, Orleans V (2021). Increased in-hospital mortality from COVID-19 in patients with schizophrenia. L’Encéphale.

[14] Nemani K, Li C, Olfson M (2021). Association of psychiatric disorders with mortality among patients with COVID-19. JAMA Psychiat.

[15] Wang Q, Xu R, Volkow ND. Increased risk of COVID‐19 infection and mortality in people with mental disorders: analysis from electronic health records in the United States - Wang - 2021 - World Psychiatry - Wiley Online Library. https://onlinelibrary.wiley.com/doi/10.1002/wps.20806. Accessed 6 July 2022. 10.1002/wps.20806 PMC7675495 33026219

[16] Clift AK, Coupland CAC, Keogh RH, Hemingway H, Hippisley-Cox J (2021). COVID-19 mortality risk in down syndrome: results from a cohort study of 8 million adults. Ann Intern Med.

[17] Emami A, Javanmardi F, Akbari A, Asadi-Pooya AA (2021). COVID-19 in patients with Down syndrome. Neurol Sci Off J Ital Neurol Soc Ital Soc Clin Neurophysiol.

[18] Hüls A, Costa ACS, Dierssen M (2021). Medical vulnerability of individuals with Down syndrome to severe COVID-19-data from the Trisomy 21 Research Society and the UK ISARIC4C survey. EClinicalMedicine.

[19] Landes SD, Turk MA, Damiani MR, Proctor P, Baier S (2021). Risk factors associated with COVID-19 outcomes among people with intellectual and developmental disabilities receiving residential services. JAMA Netw Open.

[20] Bartels SJ, Baggett TP, Freudenreich O, Bird BL (2020). COVID-19 emergency reforms in Massachusetts to support behavioral health care and reduce mortality of people with serious mental illness. Psychiatr Serv.

[21] Landes SD, Turk MA, Formica MK, McDonald KE, Stevens JD (2020). COVID-19 outcomes among people with intellectual and developmental disability living in residential group homes in New York State. Disabil Health J.

[22] Sheehan R, Dalton-Locke C, Ali A, Vera San Juan N, Totsika V, Hassiotis A. Effects of the COVID-19 pandemic on mental healthcare and services: results of a UK survey of front-line staff working with people with intellectual disability and/or autism. BJPsych Bull. 2021:1–7. 10.1192/bjb.2021.52. 10.1192/bjb.2021.52 PMC9768507 33977886

[23] Druss BG (2020). Addressing the COVID-19 pandemic in populations with serious mental illness. JAMA Psychiat.

[24] Armitage R, Nellums LB (2020). The COVID-19 response must be disability inclusive. Lancet Public Health.

[25] Shinn AK, Viron M (2020). Perspectives on the COVID-19 pandemic and individuals with serious mental illness. J Clin Psychiatry.

[26] Zimmerman S, Sloane PD, Katz PR, Kunze M, O’Neil K, Resnick B (2020). The need to include assisted living in responding to the COVID-19 pandemic. J Am Med Dir Assoc.

[27] Solis J, Franco-Paredes C, Henao-Martínez AF, Krsak M, Zimmer SM (2020). Structural vulnerability in the U.S. revealed in three waves of COVID-19. Am J Trop Med Hyg.

[28] Jenq GY, Mills JP, Malani PN (2020). Preventing COVID-19 in assisted living facilities-a balancing act. JAMA Intern Med.

[29] Walker ER, McGee RE, Druss BG (2015). Mortality in mental disorders and global disease burden implications: a systematic review and meta-analysis. JAMA Psychiat.

[30] Bartels SJ, DiMilia P (2017). Why serious mental illness should be designated a health disparity and the paradox of ethnicity. Lancet Psychiatry.

[31] Janssen EM, McGinty EE, Azrin ST, Juliano-Bult D, Daumit GL (2015). Review of the evidence: prevalence of medical conditions in the United States population with serious mental illness. Gen Hosp Psychiatry.

[32] Guan WJ, Liang WH, Zhao Y (2020). Comorbidity and its impact on 1590 patients with COVID-19 in China: a nationwide analysis. Eur Respir J.

[33] Berlin I, Thomas D, Le Faou AL, Cornuz J (2020). COVID-19 and smoking. Nicotine Tob Res Off J Soc Res Nicotine Tob.

[34] Capone GT, Chicoine B, Bulova P (2018). Co-occurring medical conditions in adults with Down syndrome: a systematic review toward the development of health care guidelines. Am J Med Genet A.

[35] Capone G, Stephens M, Santoro S (2020). Co-occurring medical conditions in adults with Down syndrome: a systematic review toward the development of health care guidelines. Part II. Am J Med Genet A.

[36] Kinnear D, Morrison J, Allan L, Henderson A, Smiley E, Cooper SA (2018). Prevalence of physical conditions and multimorbidity in a cohort of adults with intellectual disabilities with and without Down syndrome: cross-sectional study. BMJ Open.

[37] O’Leary L, Cooper SA, Hughes-McCormack L (2018). Early death and causes of death of people with intellectual disabilities: a systematic review. J Appl Res Intellect Disabil JARID.

[38] Hakim D. ‘It’s hit our front door’: homes for the disabled see a surge of Covid-19. The New York Times; 2020. https://www.nytimes.com/2020/04/08/nyregion/coronavirus-disabilities-group-homes.html. Accessed 5 Apr 2023.

[39] Levison JH, Krane D, Donelan K, et al. Best practices to reduce COVID-19 in group homes for individuals with serious mental illness and intellectual and developmental disabilities: protocol for a hybrid type 1 effectiveness-implementation cluster randomized trial. Contemp Clin Trials. 2022:107053. 10.1016/j.cct.2022.107053. 10.1016/j.cct.2022.107053 PMC9758744 36539061

[40] Baggett TP, Scott JA, Le MH (2020). Clinical outcomes, costs, and cost-effectiveness of strategies for adults experiencing sheltered homelessness during the COVID-19 pandemic. JAMA Netw Open.

[41] Losina E, Leifer V, Millham L (2021). College campuses and COVID-19 mitigation: clinical and economic value. Ann Intern Med.

[42] Neilan AM, Losina E, Bangs AC (2020). Clinical impact, costs, and cost-effectiveness of expanded severe acute respiratory syndrome coronavirus 2 testing in Massachusetts. Clin Infect Dis Off Publ Infect Dis Soc Am.

[43] COVID-19 response reporting | Mass.gov. https://www.mass.gov/info-details/covid-19-response-reporting. Accessed 2 Aug 2021.

[44] U.S. Census Bureau QuickFacts. Massachusetts. https://www.census.gov/quickfacts/MA. Accessed 21 Dec 2022.

[45] CDC. Healthcare workers. Centers for Disease Control and Prevention; 2020. https://www.cdc.gov/coronavirus/2019-ncov/hcp/planning-scenarios.html. Accessed 21 Dec 2022.

[46] Li W, Zhang B, Lu J (2020). Characteristics of household transmission of COVID-19. Clin Infect Dis.

[47] Fisher KA, Barile JP, Guerin RJ (2020). Factors associated with cloth face covering use among adults during the COVID-19 pandemic — United States, April and May 2020. MMWR Morb Mortal Wkly Rep.

[48] Polack FP, Thomas SJ, Kitchin N (2020). Safety and efficacy of the BNT162b2 mRNA Covid-19 vaccine. N Engl J Med.

[49] Kucirka LM, Lauer SA, Laeyendecker O, Boon D, Lessler J (2020). Variation in false-negative rate of reverse transcriptase polymerase chain reaction-based SARS-CoV-2 tests by time since exposure. Ann Intern Med.

[50] CDC. Health Departments. Centers for Disease Control and Prevention. 2020. https://www.cdc.gov/coronavirus/2019-ncov/php/contact-tracing/contact-tracing-plan/appendix.html. Accessed 22 Dec 2022.

[51] Massachusetts COVID-19 community mobility report. Google; 2021. https://www.gstatic.com/covid19/mobility/2021-02-02_US_Massachusetts_Mobility_Report_en.pdf.

[52] Kahn R, Holmdahl I, Reddy S, Jernigan J, Mina MJ, Slayton RB (2022). Mathematical modeling to inform vaccination strategies and testing approaches for coronavirus disease 2019 (COVID-19) in nursing homes. Clin Infect Dis.

[53] David Paltiel A, Goldie SJ, Losina E (2001). Preevaluation of clinical trial data: the case of preemptive cytomegalovirus therapy in patients with human immunodeficiency virus. Clin Infect Dis.

[54] Spies R, Siegfried N, Myers B, Grobbelaar SS (2021). Concept and development of an interactive tool for trial recruitment planning and management. Trials.

[55] Schackman BR, Scott CA, Sax PE (2007). Potential risks and benefits of HIV treatment simplification: a simulation model of a proposed clinical trial. Clin Infect Dis.

[56] Abbas I, Rovira J, Casanovas J, Greenfield T (2008). Optimal design of clinical trials with computer simulation based on results of earlier trials, illustrated with a lipodystrophy trial in HIV patients. J Biomed Inform.

[57] Lorenzana SB, Hughes MD, Grinsztejn B (2012). Genotype assays and third-line ART in resource-limited settings: a simulation and cost-effectiveness analysis of a planned clinical trial. AIDS.

[58] Brown CH, Curran G, Palinkas LA (2017). An overview of research and evaluation designs for dissemination and implementation. Annu Rev Public Health.

[59] Huang W, Chang CH, Stuart EA (2021). Agent-based modeling for implementation research: an application to tobacco smoking cessation for persons with serious mental illness. Implement Res Pract.

[60] Sheldrick RC, Cruden G, Schaefer AJ, Mackie TI (2021). Rapid-cycle systems modeling to support evidence-informed decision-making during system-wide implementation. Implement Sci Commun.

[61] Siedner MJ, Alba C, Fitzmaurice KP (2022). Cost-effectiveness of coronavirus disease 2019 vaccination in low- and middle-income countries. J Infect Dis.

[62] Reddy KP, Fitzmaurice KP, Scott JA (2021). Clinical outcomes and cost-effectiveness of COVID-19 vaccination in South Africa. Nat Commun.

[63] Reddy KP, Shebl FM, Foote JHA (2021). Cost-effectiveness of public health strategies for COVID-19 epidemic control in South Africa: a microsimulation modelling study. Lancet Glob Health.

[64] Lim TY, Stringfellow EJ, Stafford CA (2022). Modeling the evolution of the US opioid crisis for national policy development. Proc Natl Acad Sci.

