# Supplementary material for: Using simulation modeling to inform intervention and implementation selection in a rapid stakeholder-engaged hybrid effectiveness-implementation randomized trial
Source: Implement Sci Commun. 2024 Jun 24;5:70. doi: 10.1186/s43058-024-00593-w (PMC11194878; doi:10.1186/s43058-024-00593-w)
Supplement: Supplementary file 1 — Supplementary Material 1. [file 43058_2024_593_MOESM1_ESM.docx]

**Supplemental Material**

**Using Simulation Modeling to Inform Intervention and Implementation Selection in a Rapid Stakeholder-Engaged Hybrid Effectiveness-Implementation Randomized Trial**

**Becker JE *et al.***

**Additional Methods Details**

*Model Accessibility*

The Clinical and Economic Analysis of COVID-19 interventions (CEACOV) model, developed by our research team, is publicly-available at the following website: https://github.com/MGH-MPEC/CEACOV.

*Stakeholder Groups*

Stakeholders from group homes (GHs) were divided into four groups – residents of GHs, including individuals from serious mental illness (SMI) and intellectual and developmental disabilities (ID/DD) facilities; frontline staff, including direct caregivers and supervisors of individual GHs; administration, including administrative leadership from GH provider organizations; and research teams, including a group of researchers and research administrators that are part of a single provider organization. Family members of residents were also included as a stakeholder group. Finally, the larger research team, including implementation scientists, physicians, experts in survey design, biostatisticians, and data analysts, had substantial stakeholder involvement in the design and collection of data used to inform model inputs.

*Data Sources*

As described in the main text, we utilized four different types of data sources in order to populate the simulation model to reflect our population and setting of interest as accurately as possible. First, we partnered with stakeholders to obtain qualitative data from staff and residents of GHs. A survey was designed to obtain data about daily schedules, crowding, and hygiene practices for residents and staff in the GHs. Structured qualitative interviews were also designed and administered to a sample of staff and residents of the GHs. Both the survey and structured interview questions were approved by the Institutional Review Boards of MassGeneral Brigham, the Massachusetts Department of Mental Health, and the Massachusetts Department of Developmental Services. Once collected, the survey and interview data were analyzed and utilized to devise quantitative inputs reflecting averages across the sample of GHs, such as resident and staff mask adherence and contact-time between residents, staff, and the community. The second data source type was quantitative data collected through collaboration with the GH research team and administration; these data included quantitative measures obtained via the aforementioned survey, as well as group home, resident, and staff administrative and COVID-related clinical data, such as number of infections, hospitalizations, and deaths over time. These latter data were collected as part of the baseline measures for the hybrid effectiveness-implementation trial. The other data sources for model inputs were publicly available through the Massachusetts Department of Public Health and through published literature.

*Input Derivation*

Additional details of the derivations of disease natural history, infectivity, test characteristics, and other data inputs can be found elsewhere.^1-3^

Contact-hours and Infectivity

To reflect the ongoing nature of the pandemic facing the GHs, we utilized data obtained in the first year of the pandemic, reflecting closure of many resident day programs and thus increased time at home in a GH. We additionally utilized national data from the Bureau of Labor Statistics and the United States Census Bureau to quantify contact-hours in the community for staff and community members.^1,4,5^ We used a social isolation factor to adjust for pandemic conditions.^6^

Mitigation Interventions

We modeled mask efficacy by assuming a 50% reduction in disease transmission, consistent with early estimates of mask efficacy and prior studies.^1^ Mask adherence was measured through survey data collected in the GHs and, for community interactions, from the published literature.^7^ Isolation reflected GH residents isolating to their rooms and was modeled by lowering the contact-hours spent with everyone other than essential staff. Isolation for staff and community members reflected decreased contact-hours with the community, with an assumption that only 50% of individuals would adhere to isolation, as estimated based on the published literature.^8-10^ Initial vaccine uptake reflected the actual average uptake found in the participating GHs. Vaccine efficacy was modeled through a combination of full and partial immunity.^11^

**eTable 1. Breakdown of Roles of Stakeholder Groups in the Modeling Analysis.**

| Stakeholder Group | Contribution to Data Collection, Model Development, and Analysis |
| --- | --- |
| Group Home Organizations | Quantitative and qualitative data:   - Number and type of group homes - Demographics, health, and movement of residents and staff - Structure of home - Discussion of how to collect measures - Available and feasible mitigation strategies for modeling analysis   Analysis planning and iteration:   - Standing cadence to discuss and collect data - Participation in CQIC - Review of results |
| Group Home Residents | Quantitative and qualitative data:   - Movement and interactions inside and outside of group home - Feasibility of mitigation strategies   Analysis planning and iteration:   - Participation in CQIC - Review of results |
| Group Home Staff | Quantitative and qualitative data:   - Movement and interactions inside and outside of group homes - Feasibility of mitigation strategies   Analysis planning and iteration:   - Participation in CQIC - Review or results |
| Group Home Administration | Model design:   - Feasibility of mitigation strategies   Analysis planning and iteration:   - Standing cadence to discuss data - Discussing feasibility and overseeing collection of quantitative and qualitative data - Participation in CQIC - Review of initial results - Restructuring modeled mitigation strategies to reflect feasibility of implementation - Coordination among residents, family, and research team - Review of iterated results |
| Academic Research & Implementation Team | Model design, Analysis planning and iteration:   - Designating strategies for analysis based on evidence and discussions with Group Home Organizations and Group Home Administration - Implementing discussed strategies into model framework - Iterating model strategies based on standing cadence with Group Home Organizations and Group Home Administration - Participation in CQIC   Data collection:   - Designing qualitative and quantitative surveys and quantitative measures to inform data inputs - Collecting qualitative and quantitative data from all stakeholders   Model analysis:   - Interpreting collected data and delineating model input parameters - Carrying out model analysis - Presenting data to stakeholders - Iterating analysis based on feedback from all stakeholders to reflect available and feasible interventions |
| Group Home Research Team | Model design:   - Standing cadence to discuss model design   Analysis planning and iteration:   - Standing cadence to discuss data - Collaborating on survey and measure design - Collecting data across group homes and providing to academic research and implementation team - Participation in CQIC - Regular review of results |
| Group Home Residents’ Family Members | Qualitative data:   - Contributing to feasibility of interventions   Analysis planning and iteration:   - Participation in CQIC - Review of results |

**Abbreviations:** CQIC - COVID Quality Improvement Collaborative

**eFigure 1. Educational Pamphlet Drafted by Trial Team Based on Simulation Results.**

This figure shows an example of a visualization created by the trial team utilizing model-generated data to educate group home residents and staff on the anticipated impact of vaccination uptake on future infections in their particular population, given the use of data collected from the group homes for input parameter derivation. This pamphlet was used as part of the tailored best practice intervention of the hybrid effectiveness-implementation trial of COVID-mitigating practices.


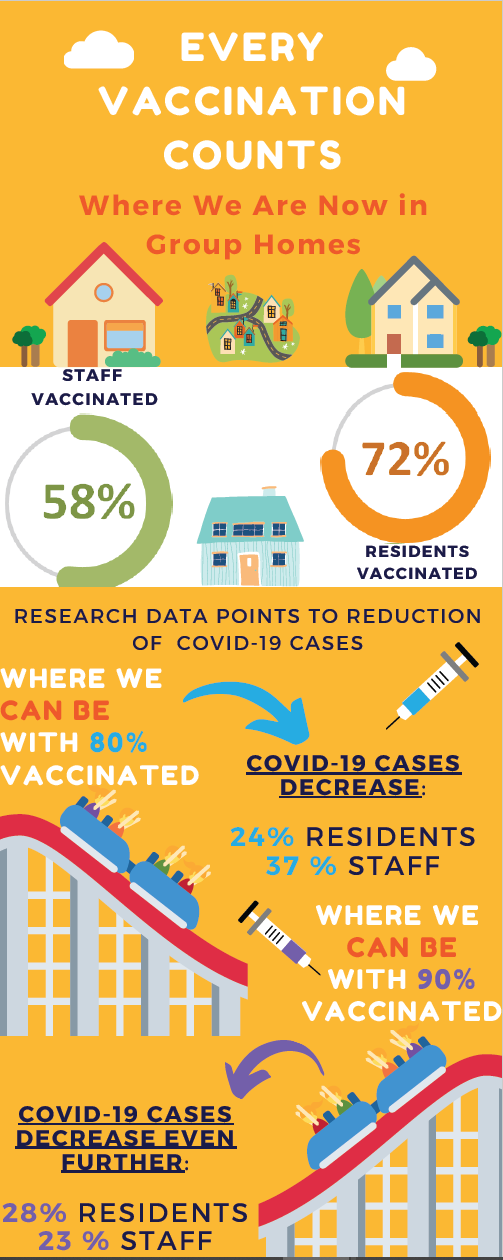


**References**

1. Losina E, Leifer V, Millham L, et al. College Campuses and COVID-19 Mitigation: Clinical and Economic Value. *Ann Intern Med*. 2021;174(4):472-483. doi:10.7326/M20-6558
2. Neilan AM, Losina E, Bangs AC, et al. Clinical Impact, Costs, and Cost-effectiveness of Expanded Severe Acute Respiratory Syndrome Coronavirus 2 Testing in Massachusetts. *Clin Infect Dis* 2020;73(9):e2908-e2917. doi:10.1093/cid/ciaa1418
3. Baggett TP, Scott JA, Le MH, et al. Clinical Outcomes, Costs, and Cost-effectiveness of Strategies for Adults Experiencing Sheltered Homelessness During the COVID-19 Pandemic. *JAMA Netw Open*. 2020;3(12):e2028195. doi:10.1001/jamanetworkopen.2020.28195
4. Databases, Tables & Calculators by Subject. *U.S. Bureau of Labor Statistics.* Available at: https://www.bls.gov/data/.
5. Historical Households Tables. *United States Census Bureau*. Available at: <https://www.census.gov/data/tables/time-series/demo/families/households.html>. Nov 2022.
6. Massachusetts Mobility changes. COVID-19 Community Mobility Report. *Google*. Available at: <https://www.gstatic.com/covid19/mobility/2021-02-02_US_Massachusetts_Mobility_Report_en.pdf>. 2 February 2021.
7. Fisher KA, Barile JP, Guerin RJ, et al. Factors Associated with Cloth Face Covering Use Among Adults During the COVID-19 Pandemic – United States, April and May 2020. *MMWR*. 2020;69(28):933-937. doi: 10.15585/mmwr.mm6928e3
8. Smith LE, Amlôt R, Lambert H, et al. Factors associated with adherence to self-isolation and lockdown measures in the UK: a cross-sectional survey. *Public Health*. 2020;182:41-52. doi: 10.1016/j.puhe.2020.07.024
9. Machida M, Nakamura I, Saito R, et al. The actual implementation status of self-isolation among Japanese workers during the COVID-19 outbreak. *Tropical Medicine and Health*. 2020;48:63. doi: 10.1186/s41182-020-00250-7
10. Bodas M and Peleg K. Self-isolation compliance in the COVID-19 era influenced by compensation: Findings from a recent survey in Israel. *Health Affairs*. 2020;39(6):926-941. doi: 10.1377/hlthaff.2020.00382
11. Reddy KP, Fitzmaurice KP, Scott JA, et al. Clinical outcomes and cost-effectiveness of COVID-19 vaccination in South Africa. *Nature Communications*. 2021;12(1):6238. doi: 10.1038/s41467-021-26557-5
